# Seasonal Variations in Game Activity Profiles and Players' Neuromuscular Performance in Collegiate Division I Basketball: Non-conference vs. Conference Tournament

**DOI:** 10.3389/fspor.2020.592705

**Published:** 2020-10-21

**Authors:** Adam J. Petway, Tomás T. Freitas, Julio Calleja-González, Lorena Torres-Ronda, Pedro E. Alcaraz

**Affiliations:** ^1^Philadelphia 76ers Athlete Care Department, Philadelphia, PA, United States; ^2^UCAM Research Center for High Performance Sport, Catholic University of Murcia, Murcia, Spain; ^3^NAR-Nucleus of High Performance in Sport, São Paulo, Brazil; ^4^Physical Education and Sport Department, Faculty of Education and Sport, University of Basque Country (UPV/EHU), Vitoria, Spain; ^5^Faculty of Kinesiology, University of Zagreb, Zagreb, Croatia; ^6^Faculty of Sport Sciences, UCAM, Catholic University of Murcia, Murcia, Spain

**Keywords:** vertical jump, RSI, game demands, fatigue, neuromuscular outputs

## Abstract

Basketball has a high demand on a player's neuromuscular system due to a high volume of explosive high-intensity actions. This study aimed to examine the seasonal variations on game demands and players' neuromuscular performance during the Non-Conference (NON-CONF) and Conference (CONF) seasons in NCAA Division I Men's Basketball. Seven NCAA Division I Basketball players' (20 ± 1.2 years, 1.95 ± 0.1 m, and 94 ± 15 kg) match activity profiles were tracked in 17 home games (7 NON-CONF; 10 CONF); furthermore, players performed a repeat hop test on a force platform the day before competition to assess neuromuscular performance. A *t*-test for paired samples was used to analyze the differences between NON-CONF and CONF. Results indicated no significant differences in Total Distance, Peak Speed, Acceleration, and Deceleration loads when comparing NON-CONF and CONF match-play. Regarding neuromuscular performance, Jump Height (*p* = 0.03; ES = 0.43) was negatively affected during CONF. Moreover, a trend toward a decline in Peak Force (*p* = 0.06; ES = 0.38) was found in CONF. Conversely, no differences were obtained regarding Reactive Strength Index and Contact Time. In conclusion, match-play demands remained constant across the season whilst neuromuscular outputs were inhibited during the CONF season.

## Introduction

Basketball is an intermittent sport in which repeated high-intensity explosive actions (i.e., jumps, accelerations, decelerations, and changes of direction) are performed during match-play (Steenland and Deddens, 1997; Calleja-González et al., 2016a,b; Svilar et al., 2018; Vázquez-Guerrero et al., 2019). Due to the force-velocity features that characterize these actions of the game, an adequate development of the neuromuscular system capabilities (i.e., strength and power) is required (Aoki et al., 2017; Edwards et al., 2018a; Ferioli et al., 2018). In fact, it has been suggested that the ability to produce high levels of force in short amounts of time is paramount and may differentiate basketballers from superior competition levels (Ziv and Lidor, 2010). For this reason, coaches and sport scientists have long been interested in the study of basketball game demands (McInnes et al., 1995; Abdelkrim et al., 2010a,b; Sampaio et al., 2015; Puente et al., 2017; Ferioli et al., 2018; Svilar et al., 2018, 2019; Vázquez-Guerrero et al., 2018, 2019) and the players' neuromuscular profile (Caterisano et al., 1997; Gonzalez et al., 2013; Edwards et al., 2018a,b; Heishman et al., 2019, 2020). A deeper knowledge on these topics could have huge implications on the global responses relative to stress imposed by competition on, for example, players' jumping or reactive strength capabilities. This is especially relevant in contexts where the season lasts for long periods and the competitive calendars are schedule-congested, as in the National Basketball Association (NBA) or college basketball competitions.

In the particular case of the National Collegiate Athletic Association (NCAA) Division I Basketball, the competitive season (where the players have to practice, compete and study) begins in November and potentially lasts up until April. There are typically 3 phases to the season: (i) the Non-Conference (NON-CONF) season, which lasts from November until December and has an inconsistent schedule and variability in competition density patterns; (ii) the Conference (CONF) schedule, held from January until early March, which is consistent in nature and has at least two competitions every calendar week; (iii) the NCAA Tournament which is played in March for teams that qualify. Despite the abundance of literature describing the demands of basketball in different levels of competition (Caterisano et al., 1997; Abdelkrim et al., 2010a; Aoki et al., 2017; McMahon et al., 2019; Souza et al., 2020), no study has focused on analyzing changes in game demands throughout the NCAA college season and the implications this could have on neuromuscular outputs.

Due to the demands and chaotic schedule of competition, it is common practice for strength and conditioning coaches and sports scientist to track and monitor neuromuscular performance outputs and fatigue throughout the competitive season (Edwards et al., 2018a,b). Understanding how these values fluctuate across the season may provide insight on how athletes are adapting to the stress imposed by the sporting activity and have a direct impact on the training loads prescribed to each athlete. In this context, previous studies from basketball and other team-sports have shown that long competitive calendars may have a detrimental effect (i.e., decreased outputs) on selected neuromuscular variables such as maximum dynamic strength, vertical jump height, or sprinting speed (Caterisano et al., 1997; Edwards et al., 2018a,b; Ferioli et al., 2018). Conversely, Gonzalez et al. (2013) observed that players who played more than ~25 min per game across an entire NBA season increased vertical jump power and improved their reaction time from pre- to post-season. Given these inconsistencies, more research is needed to better understand the fluctuation of neuromuscular performance parameters throughout the basketball season as it may provide valuable information regarding players' recovery needs and readiness to compete (McInnes et al., 1995; Abdelkrim et al., 2010b; Puente et al., 2017; Neal et al., 2018; Vázquez-Guerrero et al., 2018; Heishman et al., 2019, 2020; Svilar et al., 2019).

Considering the previous, having standardized and repeatable assessments that allow gathering information about the function of the neuromuscular system, as well as specific external load variables to the game of basketball, might be extremely relevant for trainers and staff (Cormack et al., 2008, 2013; Bishop et al., 2018). Vertical jumps, for example, have been proposed as simple monitoring tools that can be used to quantify neuromuscular fatigue, particularly through force plate evaluations (Gerodimos et al., 2008; Gathercole et al., 2015; McMahon et al., 2018). Notably, most research utilizes the countermovement jump (CMJ) as the main tool for neuromuscular fatigue evaluation in team-sports (amongst the different types of vertical jump) (Ziv and Lidor, 2010; Gonzalez et al., 2013; Gathercole et al., 2015; Suchomel et al., 2016; Edwards et al., 2018b; Neal et al., 2018). However, based on the need for rapid stretch shortening cycle actions in basketball, it may be interesting to explore a repeated-hop test to assess players readiness and fatigue levels during the competitive phase of the season (Klusemann et al., 2013). Variables obtained from this type of evaluation (e.g., peak force or reactive strength index [RSI]) can provide important information in sports that require the production of large amounts of vertical force in a short amount of time; moreover, they can reflect potential neuromuscular fatigue elicited by basketball competition (Cormack et al., 2008, 2013; Gerodimos et al., 2008; McMahon et al., 2018; Heishman et al., 2019, 2020).

To the best of authors' knowledge, no previous study has simultaneously investigated the match-play demands of NCAA Division I basketball and examined how players' neuromuscular performance, assessed through a repeated-hop test, fluctuates throughout the competitive collegiate season. From an applied standpoint, this investigation may help coaches and sport scientists design more effective training and recovery strategies (Calleja-González et al., 2016a,b) by providing insight on the effects of a basketball season on performance. Therefore, the purpose of this study was two fold: (1) to examine and compare the match demands in both a NON-CONF and CONF tournament of the NCAA Division I Men's Basketball Championship; (2) to investigate how neuromuscular performance outputs and neuromuscular fatigue levels change throughout the course of the complete collegiate basketball season.

## Materials and Methods

### Experimental Design

This descriptive longitudinal study was performed during the competitive phase of the 2017/2018 NCAA Division I collegiate basketball season. Match-play data was recorded during home games in both the NON-CONF and CONF seasons. NON-CONF occurred in the months of November and December 2017 and was classified as playing teams outside of the conference in a randomized format with a total of 12 matches (8 home and 4 away). CONF occurred during the months of January and February and was classified as playing teams within the conference with a frequency of 2 competitions per week for a total of 19 competitions (10 home and 9 away). Players' neuromuscular performance and fatigue were continuously assessed throughout the season on a weekly basis, particularly in the day before competition (i.e., Match-day−1) via a repeated-hop test. Data on each player was collected by the strength and conditioning staff as routine for the daily assessment of fatigue and player loads.

### Subjects

Seven NCAA Division I male collegiate basketball athletes (4 guards and 3 forwards; 20 ± 1.2 years, 1.95 ± 0.09 m, and 94 ±15 kg) from the same team were included in this study. The University Institutional Review Board (IRB) approved this study and researchers were provided de-identified data to analyze. By enrolling in the university's basketball program, student-athletes provided individual consent for study participation as part of their requirements as a team member. All participants were medically cleared and presented no musculoskeletal injuries or cardiovascular, respiratory, neurological, metabolic, hematological endocrine exercise disorders that might impair their performance during training or match. Additionally, no participants were using illegal drugs or taking medications, which affected body mass.

### Procedures

#### Match-Play Demands

Match-play activity profiles were tracked throughout the competitive season via spatial tracking cameras (Sport VU® Chicago, USA) (Sampaio et al., 2015; Linke et al., 2018). A total of 17 home games were analyzed during the competitive season (7 NON-CONF, 10 CONF). Six cameras were set up within the competition arena to track in-game payer loads. The primary performance variables used to track game load were Total Distance (m), Peak Speed (km·h^−1^), Acceleration and Decelerations loads expressed in arbitrary units (AU) (Vázquez-Guerrero et al., 2018; Svilar et al., 2019). Data was collected via Stats Sports Sport VU software and exported to a customized spreadsheet (Microsoft Excel 2016, USA). All seven subjects competed in each of the 17 matches.

#### Neuromuscular Testing

Each player's neuromuscular performance and fatigue were assessed on the Match-Day−1 of the 17 competitive home matches via a repeated-hop test (Flanagan and Comyns, 2008). The hop test was preceded by a standardized warm up consisting of a series of squats, lunges, and free arm swing CMJ. Three repeated-hops were performed on a triaxial force plate (9260 AA—Kistler, Switzerland) (Crewther et al., 2011). The repeat hop test was performed with the athlete's hands on their hips and after the athlete was still for a 3 s period on the force platform to stabilize body weight. Athletes were instructed to jump as high and as fast as possible 3 times with minimal ground contact time and without resetting between jumps. All tests were completed 15 min prior to practice. If the athletes did not complete the standardized warm up or the test did not fall within the 15-min window pre-practice, results were not considered (Kamonseki et al., 2018). All jumps were recorded via a data acquisition system (DAQ System Type 5691 A- Kistler, Switzerland). Each trial was exported to a text file and then imported and analyzed with the ForceDecks Software (Vald Performance, Brisbane, Australia). The primary variables examined of the 3 jumps were best Jump Height (JH) in cm, Peak force (PF) in Newtons (N), mean Contact Time (CT) of the 3 jumps in ms and best RSI (calculated by dividing JH/CT) in m·s^−1^.

### Statistical Analysis

All data was reported in mean ± SD with 95% confidence intervals (95%). Normality and homogeneity of variance were checked via the Shapiro-Wilk test (<50), revealing parametric data. Therefore, differences in performance between NON-CONF and CONF metrics were assessed by a *t*-test for paired samples. Effect sizes were calculated as Cohen's *d* (parametric data), and interpreted as trivial, <0.2; small, 0.2-0.6; moderate, 0.6-1.2 or large, 1.2-2.0 (Hopkins et al., 2009). *P* values below 0.05 were considered statistically significant (Cohen, 1988). The data was analyzed using the SPSS statistical package (version 23.0; SPSS, Inc., Chicago, IL).

## Results

Match-play activity profiles can be found in Table 1. There were no significant differences in Total Distance covered and Peak Speed achieved in competition between NON-CONF and CONF games (*p* > 0.05). Furthermore, no significant between-tournament differences were found with regards to Acceleration and Deceleration loads (*p* > 0.05).

**Table 1:** Comparison of the match-play outcomes between the non-conference and conference tournaments.

	**NON-CONF**	**CONF**	***p-value***	**ES (95% CL)**
Distance (m)	1590 ± 535	1560 ± 659	0.77	0.05 (−0.35; 0.45)
Peak Speed (km·h^−1^)	15.5 ± 1.1	15.3 ±1.4	0.53	0.13 (−0.27; 0.53)
Acceleration Load (AU)	349 ± 110	331 ± 126	0.46	0.15 (−0.25; 0.55)
Decelerations Load (AU)	643 ± 201	603 ± 235	0.31	0.18 (−0.22; 0.58)

*NON-CONF, Non-conference tournament; CONF, Conference tournament; ES, effect sizes; CL, confidence limits; AU, arbitrary units*.

**Figure 1:**
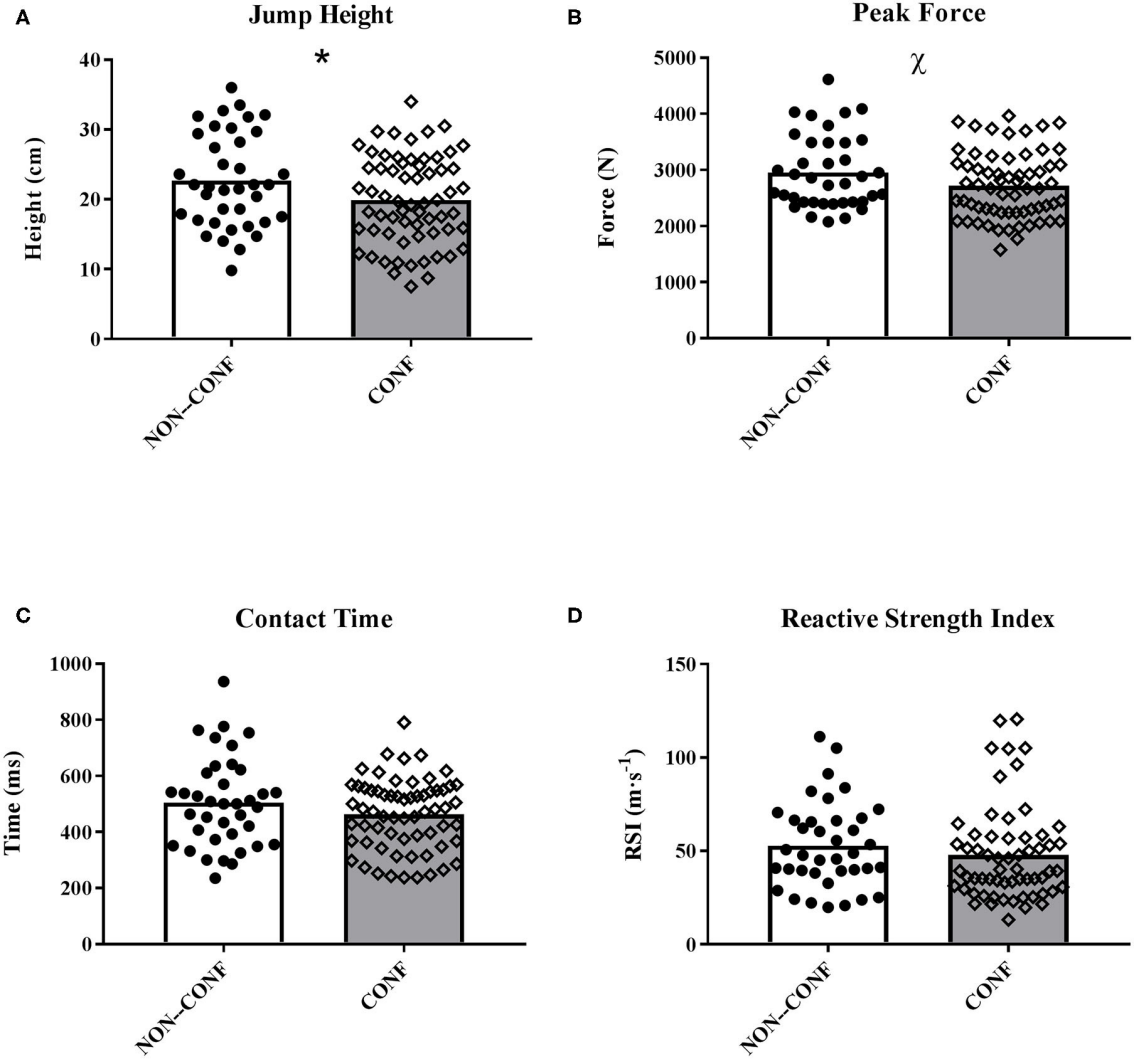
**(A)** Jump Height, **(B)** Peak Force, **(C)** Contact Time, and **(D)** Reactive Strength Index obtained on the repeated-hop test during the Non-Conference (NON-CONF) and Conference (CONF) seasons. Bars indicate mean values. The black circles and white squares represent individual data points from all the players' Match-Day−1 assessments. * Significant decrease in Jump Height. ^χ^ Trend toward decreased Peak Force.

Table 2 and Figure 1 display the neuromuscular performance outcomes. Significantly lower JH (*p* = 0.03; ES = 0.43) were observed in CONF with respect to NON-CONF. Furthermore, a trend toward a small decline in PF (*p* = 0.06; ES = 0.38) was found. Finally, no significant differences between NON-CONF and CONF were obtained for CT and RSI (*p* > 0.05).

**Table 2:** Comparison of the neuromuscular performance outcomes between Non-conference and Conference tournament.

	**NON-CONF**	**CONF**	***p-value***	**ES (95% CL)**
Jump Height (cm)	22.7 ± 6.7	19.9 ± 6.3^*^	0.03	0.43 (0.03; 0.84)
Peak Force (N)	2957 ± 651	2719 ± 596	0.06	0.38 (-0.02; 0.79)
Contact time (s)	0.50 ± 0.16	0.46 ± 0.13	0.14	0.28 (-0.12; 0.68)
RSI (m·s^−1^)	52.8 ± 23.1	48.0 ± 28.5	0.37	0.18 (-0.22; 0.58)

NON-CONF, Non-conference tournament; CONF, Conference tournament; ES, effect sizes; CL, confidence limits; RSI, Reactive Strength Index.

* *P < 0.05*.

## Discussion

The purpose of the present study was to examine and compare the game demands in both NON-CONF and CONF match-play of NCAA Division I Men's Basketball, as well as to investigate how neuromuscular performance outputs change throughout the course of the competitive collegiate basketball season (November and December 2017). The main findings from this study indicated that: (1) no difference were found in match-play demands when comparing NON-CONF to CONF seasons and (2) neuromuscular performance (i.e., JH and PF), assessed with a repeated-hop test, was negatively impacted during the CONF season. The results show that game demands appear to be constant across both competitions; nevertheless, the higher density patterns and travel characteristics of the CONF season (i.e., 19 games in ~8 weeks) may result in higher levels of residual fatigue that ultimately affect performance (McLean et al., 2010).

Previous research has examined game demands of basketball based on regular season vs. tournament competitions (Klusemann et al., 2013), different competition levels (Abdelkrim et al., 2010b; Aoki et al., 2017; Ferioli et al., 2018; Svilar et al., 2018) and playing position (Abdelkrim et al., 2010a; Puente et al., 2017; Svilar et al., 2019). However, to the authors' knowledge, no previous study has investigated the game activity profiles in elite level collegiate basketball or whether meaningful changes occur throughout the season. For example, Klusemann et al. (2013) found that the frequency of running, sprinting, and shuffling movements in seasonal games was higher than in tournament games by 8–15%, but investigated a sample U-18 youth basketball players. Conversely, the present data regarding match-play demands identified no significant fluctuations in any of the variables analyzed (i.e., Total Distance, Peak Speed, Acceleration, and Deceleration loads) when contrasting the NON-CONF and CONF seasons. These findings suggest that the activity profiles remain constant regardless of the schedule and competition characteristics in collegiate basketball format. From a practical perspective, as game loads appear to be stable throughout the competitive season, practitioners can manipulate variables outside of competition to influence performance and use this information to program typical weeks that mimic loads imposed during match-play. For example, coaches can modulate training to reflect game demands during times of the year where frequency of competition is less (i.e., NON-CONF). On the contrary, when congestion of games is high (i.e., CONF tournament) coaches may wish to limit high volumes of court transitions, accelerations, and decelerations during training to allow for an adequate recovery between consecutive matches (Abdelkrim et al., 2010a; Calleja-González et al., 2016b).

As it relates to neuromuscular performance, a distinctive aspect of the present study is that not only JH, but also other outcomes from the repeated-hop test (i.e., PF, CT and RSI) were investigated. Notably, there was a significant decrease in JH during the CONF season (Figure 1A) and a trend (*p* = 0.06) toward a decline in PF (Figure 1B). No differences were found in CT or RSI. Previous research has shown that loads imposed during training can elicit neuromuscular fatigue resulting in decreased JH and increased ground CT in elite basketball athletes (Edwards et al., 2018a,b; Heishman et al., 2019, 2020), as well as top level Australian Football (Cormack et al., 2008, 2013) and Rugby League (McLean et al., 2010) using a CMJ. Despite the CMJ being the jump test most frequently found in the scientific literature (Edwards et al., 2018a,b; Heishman et al., 2019, 2020), the repeated-hop test was used herein and, hence, direct comparisons between studies must be performed with caution. However, the rebounding aspect of a repeat-hop test has an extremely high level of specificity as it relates to the sporting activity of basketball and that is the reason why the coaching staff opted to use this assessment throughout the season. There are several potential factors that influenced the observed changes in neuromuscular performance within this present study, the first being density of games in CONF compared to NON-CONF play. In the 8-week NON-CONF season, the team was exposed to 12 games during the months of November and December (i.e., average of ~1.5 games·week^−1^). In contrast, during the 8-week cycle of the CONF season in January and February, the team completed 19 games (i.e., average of 2.4 games·week^−1^). Based on this fact, it appears that the increased frequency of games might have had a negative impact on some of the neuromuscular outputs assessed.

Further to the previous, one must also consider the travel required during different times of the year. In NON-CONF, the players only traveled via plane and stay overnight in a hotel twice. In contrast, the team had to travel 9 times during the CONF season. In this context, previous investigations have showed the detrimental effects that travel can have on performance in basketball (Steenland and Deddens, 1997). Steenland and Deddens (1997) found that less travel and more time in between competitions resulted in an improved performance in the NBA. These findings provide insight on how teams should prioritize training or recovery based on density patterns of games and travel during the competitive season. During times of less dense competitions, practitioners might want to prescribe greater volumes of resistance and strength-power related training (e.g., gym-based sessions and court-based sessions with high incidence of jumps, cuts, changes of direction) to avoid/minimize declines in neuromuscular performance later in the season. However, in match-congested moments of the season it may be more adequate to focus on more restorative training sessions to increase on-court performance (Puente et al., 2017). Based on the present research it is evident that when frequency of competition and travel demands increase practitioners should have more of an emphasis on recovery.

Notably, both peak and temporal kinetic values during jumping tasks can be useful to gain insight on the neuromuscular strategies employed for each individual athlete. RSI, assessed as a ratio of JH:CT, has been shown to be an extremely useful evaluation tool for coaches during the course of a competitive season (McMahon et al., 2018; Heishman et al., 2019). When CT increases and JH decreases, it could potentially be a sign of neuromuscular fatigue; however, when JH increases, and CT decreases this may indicate a high level of training readiness (Flanagan and Comyns, 2008; Cormack et al., 2013). In the present study, no significant differences were found in RSI, despite the decreases observed in JH. This outcome is most probably due to the small non-significant decline in CT observed. Regarding PF, this variable is another valuable force platform outcome for coaches (Gerodimos et al., 2008; Bishop et al., 2018; McMahon et al., 2018) since it has been recently recommended to be used in conjunction with JH to assess subtle differences in vertical jump performance (McMahon et al., 2019; Souza et al., 2020). In fact, both peak and time course force plate variables have been used to assess neuromuscular fatigue in athletes (Cormack et al., 2008, 2013; Gonzalez et al., 2013; Gathercole et al., 2015; Suchomel et al., 2016; Edwards et al., 2018a; McMahon et al., 2018, 2019; Neal et al., 2018; Heishman et al., 2020). Interestingly, a trend toward a small decline was found in PF when comparing CONF to NON-CONF (Figure 1B), hence supporting the notion that fatigue (or insufficient recovery) was present and vertical jump ability was affected during the more congested phase of the season. Future research is needed to gain better insight on how different metrics oscillate throughout a basketball season.

Discussion is warranted on the limitations of the present study. First, the limited sample size may have impacted the statistical analysis of the results. However, all players involved in the present research are currently in professional basketball rosters in North America and Europe, highlighting the exceptionality of the sample studied. Furthermore, it is worth emphasizing that this investigation was conducted during 16 consecutive weeks in which players were continuously assessed on a weekly basis. This is extremely difficult to accomplish in top level collegiate basketball within the constraints of limited time and resources, characteristic of applied research (Bishop, 2008). Second, match-play activity profiles were monitored only during home games due to the fact tracking system was not available at other arenas. As a consequence, potential discrepancies between the demands imposed at home vs. away games were not depicted in the present research. Finally, neuromuscular outputs may have been affected by factors other than the game and training demands in this sample of college student-athletes (i.e., academic stress, poor sleep quality, dehydration). Therefore, future research warrants the investigation of these global stressors that could have a potentially detrimental impact on performance.

## Practical Applications

The NCAA Division I Basketball schedule is demanding on student-athletes. It is imperative for practitioners working with these athletes to monitor game demands and neuromuscular outputs (and fatigue) that can blunt performance throughout the season. Having a wholistic approach allows coaches to manipulate variables outside of training to garner specific adaptations and facilitate recovery when needed. Based on the present data, no differences were found in match-play demands when comparing NON-CONF vs. CONF seasons. In contrast, neuromuscular performance (i.e., jump height and peak force) was impacted during the CONF season, when the density of games and travel requirements were higher. Understanding how these variables fluctuate during different periods of the season can have direct implications on how coaches and sports scientists' program for peak performance. From a practical perspective, when frequency of match-play is low, greater volumes of strength- and power-oriented training and on-court sessions that replicate game loads may help maintain high levels of physiological readiness. Conversely, when densities increase, the emphasis should be placed on practices that enhance and maximize recovery between games.

## Conclusion

Congestion of match-play demands can have a detrimental impact on neuromuscular outputs and impede performance. Although game demands were constant throughout the competitive season, neuromuscular profiling showed a deleterious effect based on time of year. The data highlighted the importance of load tolerance and robustness when density patterns of games are at their highest rate. These findings could potentially affect how practitioners have selective menu items to facilitate recovery vs. potentiation effects based on time of year and competition schedule.

## Data Availability Statement

The original contributions presented in the study are included in the article/supplementary material, further inquiries can be directed to the corresponding author/s.

## Ethics Statement

The studies involving human participants were reviewed and approved by The University of Arkansas. Written informed consent for participation was not required for this study in accordance with the national legislation and the institutional requirements.

## Author Contributions

AP is corresponding author and conducted the data collection. TF, PA, and JC-G all contributed to the design of the study as well as the manuscript. TF conducted the statistical analysis. LT-R was a contributing author on the manuscript. All authors contributed to the article and approved the submitted version.

## Conflict of Interest

The authors declare that the research was conducted in the absence of any commercial or financial relationships that could be construed as a potential conflict of interest.
